# Identification of Small-Molecule Inhibitors of *Babesia bovis* by High-Throughput SYBR Green I Screening

**DOI:** 10.3390/ph19091464

**Published:** 2026-09-16

**Authors:** Silviane A. Miruka, Nanang Ariefta, Azirwan Guswanto, Atefeh Fathi, Jae Seung Lee, Kota Komatsu, Keisuke Suganuma, Naoaki Yokoyama, Yoshifumi Nishikawa, Masahito Asada

**Affiliations:** 1 National Research Center for Protozoan Diseases, Obihiro University of Agriculture and Veterinary Medicine, Obihiro 080-8555, Hokkaido, Japannanang.ariefta@gmail.com (N.A.);; 2 One Health Ally Course, WISE Program for One Health Frontier Graduate School of Excellence, Hokkaido University, Sapporo 060-0818, Hokkaido, Japan

**Keywords:** *Babesia bovis*, bovine babesiosis, combination therapy, drug discovery high-throughput screening, SYBR Green I assay

## Abstract

**Background**: Bovine babesiosis, caused by *Babesia bovis*, remains a major constraint to cattle production in endemic regions, while the current control relies on only a few drugs with concerns over resistance and residues. **Methods**: This study screened 9600 chemically diverse compounds from the Drug Discovery Initiative, University of Tokyo, against *B. bovis* using a 96-well SYBR Green I high-throughput assay at 1 µM. Primary hits were subjected to dose–response testing to determine IC_50_ values, followed by Madin–Darby bovine kidney cell cytotoxicity assays for selectivity index calculation. Combination effects with diminazene aceturate were assessed using combination indices, whereby three compounds were selected for in silico ADME, drug-likeness and target prediction analyses. DDI-1101229 was further assessed by exploratory docking against BbHT1 and BbCDPK4. **Results and Conclusions**: Eight compounds showed ≥50% inhibition in the primary screen. DDI-1101229, DDI-8231037 and DDI-15106103 showed sub-micromolar activity (IC_50_ = 0.27–0.68 µM), selectivity indices > 14.7 and strong synergy with diminazene aceturate (CI < 0.4). Overall, this study identified selective, chemically diverse non-diamidine and non-imidazoline small-molecule inhibitors of *B. bovis*, highlighting alternative chemotypes with antibabesial activity.

## 1. Introduction

*Babesia bovis* is a tick-transmitted apicomplexan parasite and one of the causative agents of bovine babesiosis, which poses a threat to the livestock industry. Endemic regions include Central and South America, Australia, Africa and parts of Asia [1]. The life cycle of the parasite involves *Rhipicephalus* spp. ticks as vectors and cattle as vertebrate hosts. Following transmission by infected ticks, *B. bovis* sporozoites invade bovine red blood cells (RBCs), where they undergo asexual replication by binary fission to produce merozoites. These merozoites egress following RBC rupture and subsequently invade new RBCs [2]. The invasion and proliferation within the bovine RBCs result in acute hemolytic anemia, fever and in severe cases, sometimes neurological complications and death [1]. The erythrocytic asexual replication stage is mainly targeted by antibabesial drugs as it is the stage responsible for clinical disease [2].

Current chemotherapeutic control of bovine babesiosis relies primarily on two drugs: diminazene aceturate (DA), a diamidine compound and imidocarb dipropionate (IMDP), a carbanilide derivative. Structural molecular-modelling studies proposed that DA commonly known as berenil exacts its antiparasitic activity by binding within the DNA minor groove and interacting with AT-rich DNA sequences [3]. In contrast, the IMDP mechanism of action is incompletely resolved. The proposed mechanism of action involves inhibition of inositol entry into *Babesia*-infected erythrocytes, thus resulting in parasite starvation [4]. A report of unstable reduced susceptibility to DA in *B. bovis* over prolonged exposure to small doses has raised concerns about the long-term effectiveness of this drug [5]. In addition, imidocarb is associated with prolonged residue persistence in meat and milk thus creating food safety concerns and limiting its widespread use [6,7]. Overall, although current treatments remain useful, the potential emergence of DA-resistant *B. bovis* and the persistence of imidocarb residues highlight the need to investigate alternative compounds for bovine babesiosis.

High-throughput screening (HTS) provides a robust approach for accelerating antiparasitic drug discovery by enabling rapid evaluation of large and chemically diverse compound libraries. Fluorescence-based DNA quantification assays, particularly those using SYBR Green I, have been widely applied to intraerythrocytic parasites and allow reliable measurement of parasitemia through correlation with DNA content [8]. These assays offer a scalable and reproducible means to assess parasite growth inhibition in vitro.

To date, several anti-*B. bovis* drug candidates have been identified through in vitro compound screenings ranging from small to several hundred in scale. These studies included the use of antiprotozoal compound platforms developed for closely related protozoan parasites such as *Plasmodium* spp. [9]. The more target-based approach involved the combination of endochin-like quinolones ELQ-316 and buparvaquone, which are cytochrome *bc*1 inhibitors studied or used for *Plasmodium* spp. and *Theileria* spp. [10]. Furthermore, screenings using the U.S. Food and Drug Administration approved compounds, as well as screenings based on herbal extracts and their synthesized derivatives, have been conducted [11]. Despite these efforts, the chemical diversity of compounds systematically evaluated against *B. bovis* remains limited. To date, few studies have combined phenotypic screening of a large chemically diverse library with selectivity assessment, drug-combination analysis and exploratory in silico target evaluation. Therefore, additional screening of structurally diverse small-molecule libraries is needed to identify alternative chemotypes with antibabesial activity and to expand the chemical space available for future *B. bovis* drug-discovery studies.

In this study, a SYBR Green I-based high-throughput screening assay was used to evaluate a 9600-compound chemical library obtained from the Drug Discovery Initiative (DDI), The University of Tokyo, for inhibitory activity against *B. bovis*. The library consisted of representative small molecules selected from a larger chemical collection for pilot screening that is designed to cover structurally diverse chemical space. Primary screening outcomes were followed by dose–response analysis, cytotoxicity assessment using bovine cell lines to determine selectivity indices, evaluation of combination therapies, exploratory computational target prediction and docking analysis to provide mechanistic context for the prioritized hit compound.

## 2. Results

### 2.1. High-Throughput Screening and In Vitro Inhibitory Activity Against B. bovis

**Figure 1:**
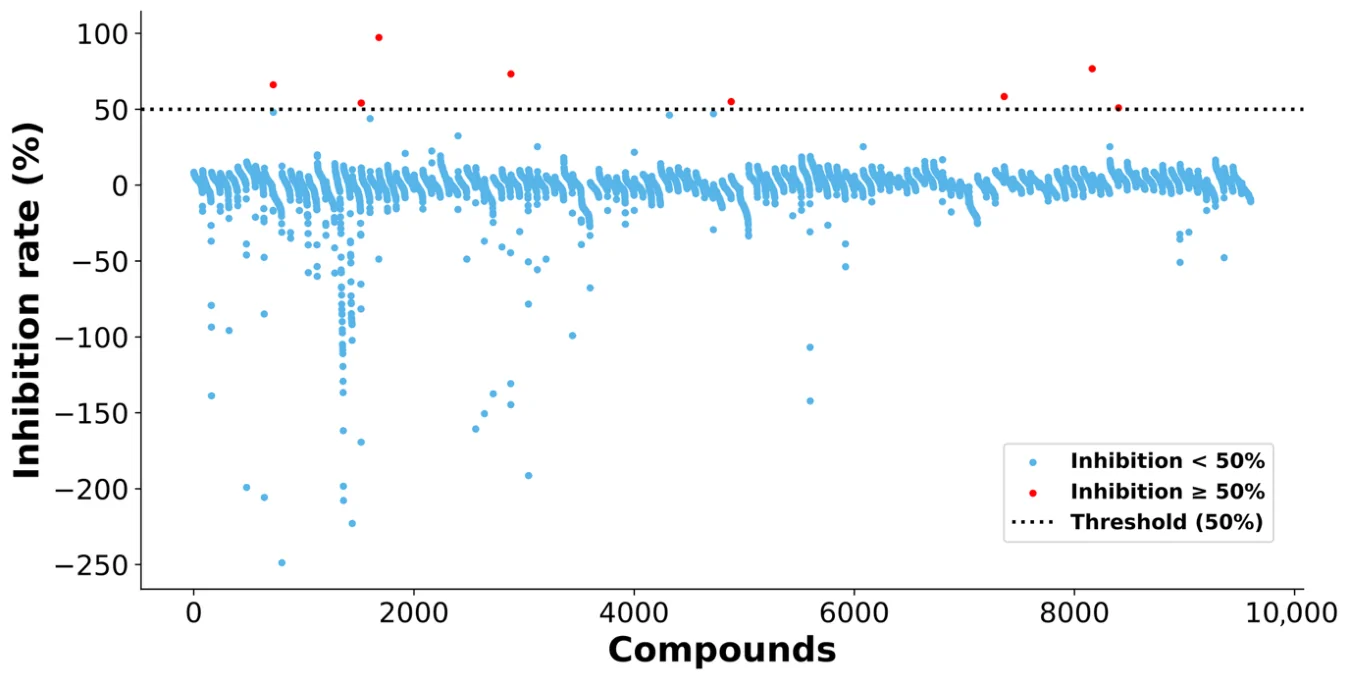
Primary screening of 9600 compounds for inhibition of *B. bovis.* Each point represents the percentage inhibition of parasite growth relative to the untreated control. Compounds producing ≥50% inhibition are highlighted in red, whereas compounds with <50% inhibition are shown in blue. The black horizontal dotted line shows the predefined hit threshold at 50% inhibition.

A total of 9600 compounds were screened for inhibitory activity against *B. bovis* at a final concentration of 1 μM across 120 plates. These compounds were obtained from DDI and comprised representative small molecules selected from a larger chemical collection for pilot screening. The assay enabled evaluation across a structurally diverse chemical space and showed a high robustness at a Z′ factor of 0.85 ± 0.11 and a strong linear correlation between parasitemia and fluorescence (R^2^ = 0.9981) (Figure S1). The Z′ factor was calculated from untreated infected erythrocyte high-signal controls and non-infected RBC low-signal/background controls. Compound-treated wells were not included in this calculation. The signal-to-background (S/B) ratio averaged 9.2 ± 3.8, with all plates exceeding the acceptable threshold (S/B > 3). Background wells exhibited a coefficient of variation (CV) of 8.19 ± 8.02%, with 82% of plates maintaining CV ≤ 10%, while control wells showed a CV of 2.76 ± 2.12%. Collectively, these performance metrics confirm the reliability of the assay for high-throughput screening. From the primary screening, eight compounds demonstrated ≥ 50% inhibition of *B. bovis* growth (Figure 1).

### 2.2. Dose–Response Profiles of Selected Compounds

Most of the compounds exhibited clear dose–response relationships, demonstrating concentration-dependent inhibition of *B. bovis* growth. The IC_50_ assay was performed on eight compounds using an eight-point, fivefold serial dilution ranging from 10 μM to 0.000128 μM, with results obtained from triplicate and three independent experiments. The IC_50_ values spanned a wide range, from 0.27 μM to greater than 10 μM, indicating substantial variability in potency among the tested compounds (Figure S2, Table 1). DDI-1101229 and DDI-8231037 showed the highest potency, with IC_50_ values of 0.27 ± 0.15 μM and 0.31 ± 0.06 μM, respectively. DDI-15106103 also demonstrated notable potency (0.68 ± 0.23 μM). For comparison, the reference drug DA exhibited an IC_50_ of 0.24 ± 0.05 μM under the same assay conditions. In contrast, DDI-1441015, DDI-226297 and DDI-1423810 exhibited moderate potency, with IC_50_ values of 1.65 ± 0.64 μM, 1.42 ± 1.88 μM and 1.91 ± 1.24 μM, respectively. DDI-1536510 and DDI-154387 displayed negligible inhibitory activity at the tested concentration, with IC_50_ > 10 μM. These results confirmed a consistent dose-dependent inhibitory effect in most of the tested compounds, while highlighting pronounced differences in efficacy. Giemsa-stained smear-based parasitemia counts provided orthogonal, non-fluorescence-based support for the SYBR Green I inhibition results. At 2 µM, a concentration corresponding to the IC_50_-based advancement cutoff and close to the 1 µM concentration used in the primary screen, all six compounds with IC_50_ values below 2 µM reduced parasitemia relative to untreated infected controls. Representative smear images and the corresponding parasitemia values are shown in Figure S3.

### 2.3. Cytotoxicity and Selectivity Index Analysis

Cytotoxicity assessment in Madin–Darby bovine kidney (MDBK) cells was performed on six compounds that had exhibited an IC_50_ < 2 μM using eight-point concentrations, fivefold serial dilution ranging from 10 μM to 0.000128 μM. Cell viability was measured using a CCK-8 assay across three independent experiments. As shown in Table 1, all six compounds had CC_50_ values greater than 10 μM, indicating that none of the compounds reduced MDBK cell viability by 50% within the tested concentration range. These findings suggest that the compounds exhibited limited cytotoxicity under the assay conditions used. The reference drug DA similarly exhibited a CC_50_ > 10 μM corresponding to an SI > 41.2. Selectivity index analysis showed that three compounds, DDI-1101229, DDI-8231037 and DDI-15106103 had favorable selectivity profiles, with SI values of >37.0, >33.3 and >14.7, respectively. In contrast, three other compounds, DDI-1441015, DDI-226297 and DDI-1423810 showed lower SI values of >6.1, >7.2 and >5.2, respectively. We selected three compounds (DDI-1101229, DDI-8231037 and DDI-15106103) for further analysis as they showed more than 10-fold selectivity between host-cell cytotoxicity and antiparasitic activity as previously suggested by Katsuno et al. [12].

### 2.4. Drug Interactions and Combination Index Analysis

The three compounds with SI > 10, DDI-1101229, DDI-8231037 and DDI-15106103, were assessed for their interactions with DA using the Chou–Talalay method. Combination index (CI) was used to show whether the interaction was synergistic, additive or antagonistic. All tested combinations yielded CI values < s1.0 at ED_50_ level indicating synergistic interactions. CI values were 0.3 ± 0.2 for DDI-1101229 + DA, 0.3 ± 0.1 for DDI-8231037 + DA and 0.4 ± 0.2 for DDI-15106103 + DA (Table 2).

### 2.5. In Silico ADME and Drug-Likeness Profiling

In silico absorption, distribution, metabolism and excretion (ADME) and drug-likeness evaluation revealed differences in the predicted physiochemical and pharmacokinetic profiles of the selected compounds (Table S1). According to Lipinski’s rule of five, drug-like compounds are expected to have a molecular weight ≤ 500 Da and CLogP ≤ 5 whereas Veber’s rule indicates a TPSA ≤ 140 Å^2^ as an acceptable threshold for oral bioavailability. DDI-8231037 had a molecular weight of 223.0 g/mol, TPSA of 50.7 Å^2^, logP of 1.1 and a high gastrointestinal absorption without violation of both Lipinski and Veber rules thus exhibiting a suitable ADME profile. DDI-1101229 also showed a comparatively suitable predicted ADME profile among the tested compounds. It showed physicochemical properties compatible with oral drug-likeness, including a molecular weight of 346.8 g/mol, TPSA of 86.8 Å^2^ and logP of 1.4. In addition, it was predicted to have high gastrointestinal absorption and compliance with both Lipinski and Veber rules. However, DDI-1101229 was predicted to inhibit CYP1A2, which indicates a potential metabolism-related liability. In contrast, DDI-15106103 exhibited some properties associated with reduced oral drug-likeness, high polarity (TPSA = 182.1 Å^2^) and reduced predicted gastrointestinal absorption. This compound failed the Veber criteria (TPSA > 140) and was predicted to inhibit CYP3A4, which may indicate a higher risk of poor absorption and potential drug–drug interaction liability. No PAINS alerts were predicted for the tested compounds, showing that none of the compounds contained any structural feature associated with pan-assay interference compounds. Overall, DDI-8231037 and DDI-1101229 showed comparatively more suitable predicted drug-like and ADME profiles than DDI-15106103. The reference antibabesial drugs DA and IMDP showed less favorable predicted oral drug-likeness profiles compared with DDI-8231037 and DDI-1101229. Both DA and IMDP had low predicted gastrointestinal absorption, high TPSA values above the Veber threshold of 140 Å^2^, and two Lipinski violations related to high numbers of nitrogen or oxygen atoms and NH or OH groups. DA also showed one PAINS alert corresponding to an azo-type structural alert, whereas IMDP showed no PAINS alert. Neither DA nor IMDP was predicted to inhibit CYP enzymes based on the CYP inhibition analysis.

### 2.6. Target Prediction for DDI-1101229

Since DDI-8231037 and DDI-15106103 contained undefined stereochemical features, target prediction and docking were only performed on DDI-1101229. Solute carrier family 2 member 1 (SLC2A1) and solute carrier family 2 member 3 (SLC2A3) were the highest-ranked targets, each with a prediction probability of 0.799, followed by solute carrier family 2 member 2 (SLC2A2) at 0.749 (Table S2). Kinase-associated predictions occurred at lower ranks and included epidermal growth factor receptor (EGFR), ribosomal protein S6 kinase B1 (RPS6KB1), mechanistic target of rapamycin (MTOR), phosphatidylinositol-4,5-bisphosphate 3-kinase catalytic subunit alpha (PIK3CA) and erb-b2 receptor tyrosine kinase 2 (ERBB2). The dominance of solute carrier family 2 (SLC2) transporters supported selection of *Babesia bovis* hexose transporter 1 (BbHT1) as a predicted target for DDI-1101229. In addition to SLC2 transporters, human kinases appeared on the lower ranks of the proteins predicted to be targeted by DDI-1101229; hence *Babesia bovis* calcium-dependent protein kinase 4 (BbCDPK4) was chosen as a potential DDI-1101229 target in *B. bovis*. Moreover, it was previously shown that bumped kinase inhibitors impair *B. bovis* egress [13], thus supporting kinase-mediated egress as a relevant drug-targeting process. BbCDPK4 was not inferred as a direct orthologue of a specific predicted human kinase.

### 2.7. Structural Models of Bbht1 and Bbcdpk4

The selected BbHT1 model reproduced an inward-open transporter cavity and had a mean pocket Predicted Local Distance Difference Test (pLDDT) of 91.4, pocket predicted aligned error (PAE) of 2.56 and conserved-cavity anchor root-mean-square deviation (RMSD) of 2.96 Å relative to GLUT1. The modest 29% template identity nevertheless requires caution when interpreting individual residue positions. The selected BbCDPK4 model had a mean adenosine triphosphate (ATP)-pocket pLDDT of 89.1. It contained the small Thr141 gatekeeper, an intact Asp208–Phe209–Gly210 aspartate–phenylalanine–glycine (DFG) motif and the conserved Lys93–Glu112 catalytic ion pair at 3.57 Å (Table S3). Together these supports an active docking-competent kinase conformation.

### 2.8. Docking and Interaction Simulation of DDI-1101229 with Babesia Proteins

**Figure 2:**
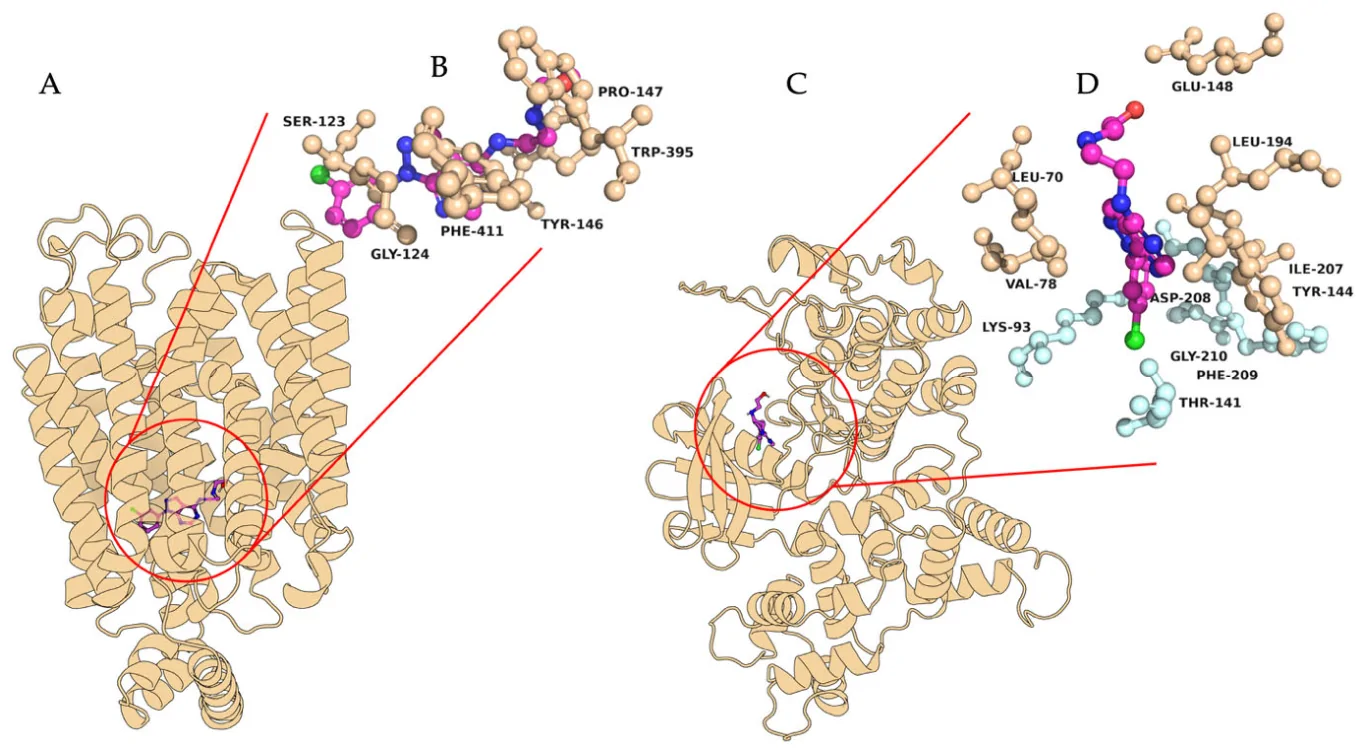
Predicted docking poses of DDI-1101229 in BbHT1 and BbCDPK4. (**A**) Overall structure of the predicted BbHT1 model showing compound DDI-1101229 positioned in the central cavity. (**B**) Enlarged view of the BbHT1 pocket showing predicted interactions with Ser123, Gly124, Tyr146, Pro147, Trp395 and Phe411. (**C**) Overall structure of the predicted BbCDPK4 model showing compound DDI-1101229 in the ATP-binding pocket. (**D**) Enlarged view showing predicted interactions with Leu70, Val78, Tyr144, Glu148, Leu194 and Ile207. The catalytic Lys93, Thr141 gatekeeper and DFG motif (Asp208–Phe209–Gly210) are shown in cyan as structural landmarks. Protein models and directly interacting residues are shown in tan, whereas compound DDI-1101229 is shown in magenta. Protein–ligand interactions were identified using PLIP and residues are numbered according to the respective *B. bovis* protein sequences.

The highest convolutional neural network (CNN)-scoring BbHT1 pose was obtained from representative conformer 2 of DDI-1101229. The displayed pose had an affinity of −7.86 kcal/mol and CNN pose score of 0.835; the best affinity among all rep2 poses was −8.26 kcal/mol (Table S4). The terminal aminoalcohol formed hydrogen bonds with Ser123 and Gly124, while Tyr146 formed two predicted hydrogen bonds. Hydrophobic contacts involved Phe19, Tyr146, Pro147, Trp395 and Phe411 (Figure 2). The highest CNN-scoring BbCDPK4 pose was obtained from representative conformer 3. The displayed pose had an affinity of −7.19 kcal/mol and CNN pose score of 0.960; the best affinity among all rep3 poses was −7.32 kcal/mol (Table S4). Predicted hydrogen bonds involved the backbones of Leu70 and Tyr144 and the side chain of Glu148 (Figure 2). Hydrophobic contacts were assigned to Val78, Leu194, and Ile207. However, DDI-1101229 did not produce a direct PLIP-defined contact with the Thr141 gatekeeper.

Comparator docking showed that compound **49** docked to BbHT1 with a highest-CNN-pose affinity of −9.99 kcal/mol, CNN pose score of 0.665 and best affinity among nine poses of −10.81 kcal/mol, with predicted contacts involving Phe19, Trp395, Phe411, Thr415, and Trp419 (Table S6). BKI-1294 docked to BbCDPK4 with a highest-CNN-pose affinity of −8.88 kcal/mol, CNN pose score of 0.860, and best affinity among nine poses of −9.38 kcal/mol, with predicted contacts involving Tyr144, Thr141, Val78, Lys93, Leu194, Ile207, Asp208, and Phe209 (Tables S5 and S7).

## 3. Discussion

The present study describes the implementation of a SYBR Green I-based HTS platform for identification of inhibitors against *B. bovis*. The assay demonstrated strong performance metrics across 120 plates, with a high Z′ factor (0.85 ± 0.11) and a strong linear correlation between parasitemia and fluorescence (R^2^ = 0.9981). The S/B ratio was 9.2 ± 3.8, with all plates exceeding the minimum acceptable threshold (S/B > 3), indicating adequate signal separation despite inter-plate variability. The observed variation in S/B values is likely attributable to differences in signal intensity between plates rather than assay instability, as supported by the low variability in control wells and acceptable coefficients of variation in background wells. The coefficient of variation for background (CV minimum) wells was 8.19 ± 8.02%, with 82% of plates exhibiting CV ≤ 10%.

Control wells demonstrated low variability (CV maximum = 2.76 ± 2.12%), supporting the overall precision and reliability of the assay. These parameters meet commonly accepted thresholds for HTS assay quality (Z′ ≥ 0.5), supporting the robustness and reproducibility of the platform [14]. Although DA was not included as a per-plate positive inhibition control during the primary HTS, it was included as a reference drug during IC_50_ confirmation. Screening of 9600 compounds yielded eight hits with ≥ 50% inhibition at a final concentration of 1 µM, corresponding to a hit rate of approximately 0.08%. This low hit rate is primarily because the DDI pilot library was chemically diverse and was not specifically enriched for antibabesial activity, which may have further limited the number of active compounds identified. Nevertheless, the low hit rate is consistent with stringent antiparasitic screening experiments. For example, Dechering et al. screened 141,786 compounds from the MMV Hit Generation Library 1 against asexual blood-stage *Plasmodium falciparum* where they identified 75 screening-active compounds, corresponding to a hit rate of 0.05% [15]. Similarly, a high-throughput screen targeting the *Toxoplasma gondii* rhoptry kinase ROP18 reported a hit rate of approximately 0.04% [16]. Together, these examples indicate that low hit recovery rates can occur in stringent antiparasitic and parasite-related screening experiments. Dose–response analysis identified three compounds with sub-micromolar activity, with IC_50_ values ranging from 0.27 to 0.68 µM. In parallel, DA produced an IC_50_ of 0.24 ± 0.05 µM under the same assay conditions. Thus, the most active hit compounds showed inhibitory activity in a range comparable to the reference drug within the present experimental system. The DA IC_50_ value in this study falls within the range of previously reported in vitro DA activity against *B. bovis*, which varies from approximately 0.08 to 0.75 µM depending on assay format [5,17]. Because SYBR Green I is a fluorescence-based readout, compound-dependent fluorescence interference, including quenching or autofluorescence, cannot be fully excluded from fluorescence-based screening alone. Previously a study on validation of the SYBR Green I assay for *B. bovis* showed strong agreement between fluorescence-based measurements and microscopy-based parasitemia determination using Giemsa-stained smears [8]. Therefore, Giemsa-stained smear-based parasitemia counting was used in the present study as a non-fluorescence-based confirmation for the six compounds with IC_50_ values below 2 µM. The reduced parasitemia observed in microscopic examination of compound-treated cultures therefore reflects true *B. bovis* growth inhibition rather than fluorescence quenching by the compounds. Altogether, the results observed from DA response and Giemsa staining in this present study further support the reliability of the assay, thus providing an internal reference for interpreting the potency of the identified hits.

The selected hit compounds also showed SI values above the commonly used early-stage antiparasitic threshold of 10, DDI-1101229 (SI > 37.0), DDI-8231037 (SI > 33.3) and DDI-15106103 (SI > 14.7), supporting their prioritization for further evaluation as previously described by Katsuno et al. [12]. While this threshold is widely applied as an initial benchmark, higher selectivity margins may be required depending on the intended application.

In addition to potency and selectivity, a qualitative structural comparison was carried out between the three compounds which exhibited selectivity indices greater than 10 (DDI-8231037, DDI-1101229 and DDI-15106103) and the currently used antibabesial drugs (DA and IMDP). This comparison provided further insight into the structural distinction of the three selected compounds from the established drugs. Diminazene contains a symmetrical bis-amidinophenyl structure connected through a triazene linker, whereas imidocarb possesses a symmetrical diaryl urea scaffold bearing two imidazoline groups. Both established drugs therefore contain two strongly basic amidine-type centers and are expected to exist predominantly as cationic species under physiological conditions. In contrast, the three identified compounds were structurally asymmetric and lacked the characteristic paired amidine or imidazoline groups. DDI-1101229 contained a nitrogen-rich fused heteroaromatic core, a chlorophenyl group and a flexible diamino-alcohol side chain. Although it shared the presence of aromatic and basic nitrogen-containing regions with diminazene and imidocarb, its overall scaffold was distinct. In broad comparison with other antiprotozoal compounds, DDI-1101229 may be compared with N-CBZ, an aminoalcohol-carbazole compound, as both contain aromatic/fused-ring character and basic aminoalcohol functionality. In a previous study, N-CBZ was identified as a *P. falciparum* Hsp90-selective inhibitor and it showed that N-CBZ derivatives were more likely to inhibit *P. falciparum* growth when they bound PfHsp90 [18]. This comparison suggests that aminoalcohol-containing aromatic chemotypes can inhibit intraerythrocytic protozoan parasites through defined protein-associated mechanisms. However, the PfHsp90 mechanism cannot be directly extrapolated to *B. bovis* and DDI-1101229 was not evaluated against BbHsp90 in the present study.

DDI-8231037 possessed a smaller dimethoxy-substituted tetrahydroisoquinoline-like scaffold bearing hydroxymethyl substituents and contained only one secondary amine as its only basic center. Its constitution corresponds to calycotomine. Although anti-*Babesia* activity of calycotomine has not been identified, tetrahydroisoquinoline-containing chemotypes have been shown to inhibit intraerythrocytic protozoan parasites. Spirofused tetrahydroisoquinoline–oxindole hybrids were previously reported to be fast-acting antimalarial agents, with moxiquindole showing activity against asexual blood-stage *P. falciparum*, including chloroquine-sensitive and multidrug-resistant strains. Mechanistic investigation suggested that moxiquindole inhibited merozoite egress, hemoglobin degradation and vacuolar lipid dynamics [19]. However, these mechanisms cannot be directly extrapolated to *B. bovis*, because *Babesia* spp. differ from *Plasmodium* in hemoglobin digestion and do not form hemozoin. Therefore, the activity of DDI-8231037 in the present study should be interpreted as chemotype-level support for tetrahydroisoquinoline-related scaffolds as potential intraerythrocytic antiprotozoal hits, while its direct target in *B. bovis* remains to be determined.

DDI-15106103 was the most structurally complex compound, as it incorporated an amide-substituted thiophene, a methoxyphenyl group, a methylpiperidine sulfonamide, an ester linkage and two amide functionalities, without the symmetrical dicationic architecture of the established drugs. DDI-15106103 may be compared at the chemotype-feature level with sulfonamide-containing apicomplexan inhibitors such as KNX-115. Although DDI-15106103 is not a direct analogue of KNX-115 and the two compounds do not share the same complete scaffold, this comparison is useful because both are sulfonamide-containing small molecules evaluated in apicomplexan parasite drug-discovery contexts. KNX-115 was reported as a potent inhibitor of *P. falciparum* myosin A, PfMyoA, an essential class XIV myosin motor involved in parasite motility and host-cell invasion. KNX-115 inhibited actin-activated PfMyoA ATPase activity and blocked *P. falciparum* asexual blood-stage growth [20]. This comparison indicates that sulfonamide-containing small-molecule chemotypes can inhibit apicomplexan parasites through a defined cytoskeletal protein target. However, whether this mechanism applies to *B. bovis* remains unclear, as DDI-15106103 was not tested against BbMyoA or related glideosome-associated proteins. Altogether, these comparisons suggest that the identified compounds are chemically distinct from current antibabesial drugs, yet they contain structural features that were previously associated with antiprotozoal activity in other studies.

Since the three active compounds do not share a common core scaffold, a conventional structure–activity relationship analysis could not be performed in this study. Nevertheless, their whole-cell activities suggest that inhibition of *B. bovis* can occur across chemically diverse scaffolds containing different combinations of aromatic, potentially ionizable and hydrogen-bonding functionalities. This indicates that alternative structural motifs beyond the classical amidine-containing or imidazoline-containing antibabesial drugs may support activity against *B. bovis*.

In addition to structural novelty and predicted developability, exploratory target prediction and docking were performed to generate preliminary mechanistic hypotheses. Among the three selected hits, DDI-1101229 was prioritized for exploratory docking because it had a defined overall structure and acceptable predicted physicochemical properties. In contrast, DDI-8231037 and DDI-15106103 were not included in docking because their submitted structural representations contained undefined stereochemical features, which could affect generated three-dimensional conformers and complicate interpretation of docking poses and scores. BbHT1 has been functionally characterized as a *B. bovis* hexose transporter [21] and its selection was supported by the predominance of human SLC2 transporters in the ligand-based prediction. The predicted hydrogen-bond network and contacts with aromatic cavity residues, including Trp395 and Phe411, are compatible with occupation of the transporter cavity. This may suggest that inhibition of hexose uptake and consequent disruption of parasite energy metabolism may represent one possible mechanism of action for compound DDI-1101229. However, the comparatively low template identity and conformational flexibility of sugar transporters limit residue-level certainty. BbCDPK4 was selected through a separate biological rationale; its high template identity and conserved kinase-pocket features provide stronger structural support for the model. DDI-1101229 was also predicted to occupy the ATP-binding cleft of BbCDPK4 and form a hinge-region interaction with Tyr144, suggesting potential inhibition of its kinase activity. In a previous study it was shown that BKIs that were evaluated against apicomplexan could target PfCDPK4 thus blocking parasite development, while BKI RM-1-152 was shown to inhibit *B. bovis* merozoite egress [13]. For docking comparison, compound **49** and BKI-1294 showed stronger predicted affinities toward BbHT1 and BbCDPK4, respectively, thus supporting that the predicted binding pockets could accommodate known comparator ligands. The different receptor classes, model uncertainties and docking search spaces further limit cross-target comparison (Tables S6 and S7, respectively). The results should therefore be interpreted as structural hypotheses that can guide future glucose-uptake or biochemical kinase-inhibition. No target validation was performed in the present study.

In silico ADME analysis was used to evaluate physicochemical and pharmacokinetic properties relevant to compound developability, including predicted absorption-related behavior and metabolic profile. DDI-8231037 exhibited a low molecular weight (223.0 g/mol), low polarity (TPSA = 50.7 Å^2^), and moderate lipophilicity (logP = 1.1), which fall within commonly accepted ranges associated with oral drug-likeness and predicted absorption-related properties. DDI-1101229 similarly exhibited physicochemical parameters within these ranges, including a molecular weight of 346.8 g/mol, TPSA of 86.8 Å^2^ and logP of 1.4, along with predicted high gastrointestinal absorption. Although DDI-1101229 was predicted to inhibit CYP1A2, its overall profile remained favorable compared with DDI-15106103, DA and IMDP. In contrast, DDI-15106103 displayed characteristics associated with reduced oral bioavailability, including higher molecular weight (495.6 g/mol), elevated polarity (TPSA = 182.1 Å^2^), low predicted gastrointestinal absorption and predicted CYP3A4 inhibition, which may indicate a higher risk of metabolic liabilities and drug–drug interactions. For comparison, the reference antibabesial drugs DA and IMDP also showed low predicted gastrointestinal absorption, high polarity, Veber rule violations due to TPSA > 140 Å^2^ and lower bioavailability scores (0.2) than the three identified compounds (0.6). DA and IMDP also showed Lipinski violations related to high numbers of nitrogen/oxygen atoms and hydrogen-bond donor groups, whereas the three identified compounds showed no Lipinski violations. In addition, DA showed one PAINS alert, whereas the three identified compounds and IMDP showed no PAINS alerts. These findings suggest that DDI-8231037 and DDI-1101229 may have comparatively suitable predicted pharmacokinetic profiles for further development, whereas DDI-15106103 may require further structural optimization despite its biological activity. In silico ADME prediction tools, such as SwissADME, provide early-stage assessment of drug-likeness and pharmacokinetic properties in drug discovery pipelines [22].

This study also identified synergistic interactions between the selected compounds and DA, where all combinations exhibited CI values < 1.0. According to the the Chou–Talalay method, CI values < 1.0 indicate synergism, CI = 1.0 indicates an additive effect, and CI > 1.0 indicates antagonism [23]. Although reduced effective concentrations were observed in vitro, the implications of these interactions for resistance development or residue levels in food-producing animals remain uncertain. Drug combinations can influence pharmacokinetics, toxicity and selection pressure in complex ways and these effects require validation in the appropriate in vivo models.

Despite these promising findings, the present study has a few limitations. For instance, the identified compounds were evaluated using in vitro growth inhibition and cytotoxicity assays, hence their in vivo efficacy, pharmacokinetic behavior and safety remain to be determined. The MDBK cytotoxicity assay provided only an initial mammalian safety assessment and did not specifically evaluate mitochondrial toxicity. Therefore, mitochondrial liability testing, such as evaluating HepG2 cells cultured in galactose-containing medium, could be considered in future studies. The mechanism-of-action was not verified and the parasite targets responsible for the observed activity remain to be confirmed. Lastly, the compounds were not evaluated against DA-reduced-susceptibility *B. bovis* lines; hence potential cross-resistance with DA cannot be excluded and remains to be determined in future studies.

## 4. Materials and Methods

### 4.1. Parasite Culture

*Babesia bovis* (Texas strain) was maintained in continuous microaerophilic culture as previously described [24]. The parasites were propagated in GIT medium (FUJIFILM Wako Pure Chemical Corporation, Osaka, Japan) supplemented with 10% bovine RBCs (Japan Bio Serum Co., Ltd., Fukuyama, Japan) at 37 °C in a controlled gas environment (5% CO_2_, 5% O_2_, 90% N_2_). Cultures were monitored microscopically on Giemsa-stained smears and parasitemia was routinely adjusted to 1% using uninfected RBCs.

### 4.2. Compound Library

A collection of 9600 chemically diverse small molecules, obtained from DDI—The University of Tokyo, was screened. The compounds were supplied in pre-spotted 96-well plates and were tested at a final working concentration of 1 µM. All plates were kept at −30 °C and allowed to equilibrate to room temperature for approximately 30 min to 45 min prior to the assay.

### 4.3. Growth Inhibitory In Vitro Assay

The in vitro inhibitory activities were evaluated using SYBR Green I based fluorescence assay as previously described for *Babesia* parasites [8,25]. All compounds were initially screened at a final concentration of 1 µM in black, flat-bottom 96-well plates (Greiner Bio-One GmbH, Kremsmünster, Austria). Each well contained 100 µL of *B. bovis* culture adjusted to 1% parasitemia and 10% hematocrit. Wells containing non-infected RBCs and untreated infected cultures served as the low-signal/background and high-signal controls, respectively. For the primary screen, the Z′ factor was calculated using untreated infected cultures as the high-signal control and wells containing non-infected RBCs as the low-signal/background control, according to the following equation: Z′ = 1 − [3(SDhigh + SDlow)/|Meanhigh − Meanlow|]. Compound-treated wells were excluded from the Z′ factor calculation. Thus, the Z′ factor represented the assay window between parasite-associated fluorescence and RBC/background fluorescence. Plates were incubated for 48 h under microaerophilic conditions without medium change. After drug exposure, parasite DNA content was quantified using a modified SYBR Green I lysis method. Briefly, 100 µL of lysis buffer (130 mM Tris-HCl, 10 mM EDTA, 0.016% saponin, 1.6% Triton X-100) containing 2× SYBR Green I dye (Lonza Walkersville, Inc., Walkersville, MD, USA; 10,000×) was added directly to each well. Plates were gently mixed and incubated in the dark for 1 h at room temperature before fluorescence measurement at 485 nm excitation and 535 nm emission using a SpectraMax^®^ M5 microplate reader (Molecular Devices, LLC, San Jose, CA, USA). Parasite growth inhibition was calculated from normalized fluorescence values. The primary HTS was performed as a single screen using pre-spotted compound plates with a fixed layout. Compounds showing ≥ 50% inhibition were selected for subsequent confirmatory dose–response analysis. These selected compounds were tested in eight-point, five-fold serial dilutions beginning at 10 µM to 0.000128 µM. The experiment was carried out in the same manner as in growth inhibitory assay and IC_50_ values were calculated from fluorescence data using nonlinear dose–response regression (log(inhibitor) vs. response) in GraphPad Prism 9 (GraphPad Software, San Diego, CA, USA). Each concentration was tested in triplicate and assays were performed in at least three independent biological experiments. DA was not included as a per-plate positive inhibition control during the primary screening; however, DA was evaluated as a reference drug under the same assay conditions to determine its IC_50_ against *B. bovis*. Compounds exhibiting an IC_50_ value below 2 µM were subsequently advanced for evaluation in cytotoxicity assays. To further assess parasite growth inhibition independently of SYBR Green I fluorescence, Giemsa-stained thin blood smears were prepared from treated and untreated *B. bovis* cultures after 48 h of incubation. Smears were fixed with methanol (Nacalai Tesque, Kyoto, Japan), stained with Giemsa solution (Sigma-Aldrich, St. Louis, MO, USA) and examined by light microscopy. Parasitemia was determined by counting infected erythrocytes among at least 1000 RBCs per smear and compared with untreated infected controls.

### 4.4. Cytotoxicity Assay and Selectivity Index Analysis

The in vitro cytotoxicity of the selected six compounds, DDI-1101229, DDI-8231037, DDI-1441015, DDI-15106103, DDI-226297 and DDI-1423810 was evaluated using Madin–Darby bovine kidney (MDBK) cells in accordance with the recommendation for cell counting Kit-8 (CCK-8; Dojindo Laboratories, Kumamoto, Japan). The MDBK cells were seeded into 96-well tissue culture-treated plates (TPP Techno Plastic Products AG, Trasadingen, Switzerland) at a density of 5 × 10^4^ cells/mL, dispensing 100 µL per well. Plates were incubated for 24 h at 37 °C in a humidified atmosphere containing 5% CO_2_ and 5% O_2_ to allow cell adherence.

After the adherence period, 100 µL of culture medium containing serially diluted compounds was added to the wells. In this study, each compound was tested in eight-point, five-fold serial dilutions beginning from 10 µM to 0.000128 µM final concentration, mirroring concentrations used in IC_50_ determination in *B. bovis* assays. Treatments, vehicle controls and blank wells were arranged in a predetermined assay layout. The outer left and right columns of the 96-well plates were filled with medium and were not used for treatment conditions to minimize potential edge effects. Each compound concentration was tested in triplicate wells, and cytotoxicity assays were performed in three independent experiments. Cells were then incubated with the compounds for 48 h at 37 °C and 5% CO_2_.

After drug exposure, 10 µL of CCK-8 reagent was added to each well, and plates were incubated for an additional 3 h at 37 °C. Absorbance values were measured at 450 nm using a SpectraMax^®^ M5 microplate reader. Wells containing untreated cells served as the negative control, while wells containing medium without cells served as blanks. Cell viability (%) was calculated relative to untreated controls, whereas cytotoxic concentration values (CC_50_) were generated using nonlinear dose–response regression (log(inhibitor) vs. response). DA was also evaluated as a reference drug under the same cytotoxicity assay conditions to determine its CC_50_. The selectivity index (SI) was calculated for each compound as the ratio of host-cell cytotoxicity to antiparasitic activity using the following formula: SI = CC_50_ (MDBK cells)/IC_50_ (*B. bovis*).

### 4.5. In Vitro Drug Combination Assays

Drug-combination studies were performed for three selected compounds, DDI-8231037, DDI-1101229 and DDI-15106103, in combination with DA (Sigma-Aldrich, MA, USA). Each compound was tested both as monotherapy and in fixed-ratio combinations with DA. Combination concentrations were selected based on the individual IC_50_ values of each compound and DA. The IC_50_ value of DA against *B. bovis* Texas strain was 0.23 µM. The three compounds and DA were tested at 4×, 2×, 1×, 0.5× and 0.25× of their respective IC_50_ values. At least three independent biological combination assay experiments were conducted under similar experimental conditions as the dose–response experiments using the SYBR Green I assay (see Section 4.3). Fractional inhibition data were obtained for each concentration and combination. Drug interaction effects were evaluated using the Chou–Talalay method as implemented in CompuSyn software (version 1.0; ComboSyn, Inc., Paramus, NJ, USA) [23]. The fraction affected (Fa), representing the proportion of parasite growth inhibited at each drug concentration, was calculated from normalized fluorescence values. Dose effect parameters were derived from median-effect plots generated from monotherapy dose–response data according to the median effect principle. For descriptive classification, combination index (CI) of 1.0 was considered additive, whereas CI values < 1.0 were considered synergistic interactions.

### 4.6. In Silico ADME and Drug-Likeness Analysis

ADME properties, as well as drug-likeness characteristics of the selected compounds, DDI-8231037, DDI-1101229 and DDI-15106103 alongside DA and IMDP, were predicted using web-based tools SwissADME and pkCSM. The canonical simplified molecular input line entry system (SMILES) was used as input for each compound. Physicochemical parameters, including molecular weight, lipophilicity (logP), hydrogen bond donors and acceptors and topological polar surface area (TPSA) were calculated. Drug-likeness was evaluated based on Lipinski’s rule of five as well as Veber criteria associated with oral bioavailability [26]. Pharmacokinetic properties, including gastrointestinal (GI) absorption was predicted using the SwissADME web platform [22], while cytochrome P450 (CYP) enzyme inhibition was predicted using the pkCSM web server [27]. In addition, medicinal chemistry filters, such as pan-assay interference compound (PAINS) alerts, were applied in order to identify potentially problematic compound substructures [26,27]. These predictions were used to assess the pharmacokinetic suitability and developability of the identified compounds.

### 4.7. Computational Target Prediction, Protein Modelling and Molecular Docking

#### 4.7.1. Chemical Structure and Stereochemistry Assessment

Prior to computational target analysis, the SMILES structures of the three selected hit compounds were examined. The stereochemistry was considered undefined when stereochemical notation was absent from the submitted SMILES. DDI-8231037 and DDI-15106103 contained undefined stereochemical features; therefore target prediction and docking were restricted to DDI-1101229, which had the most suitable overall structure for exploratory target analysis.

#### 4.7.2. Target Prediction and Target Selection

Target prediction was restricted to DDI-1101229 and performed using SwissTargetPrediction web platform in the *Homo sapiens* setting [28], because *B. bovis*-specific target prediction is not supported by this platform and experimentally annotated ligand–target information for *B. bovis* remains limited. Predicted human targets were ranked using the default combined two-dimensional and three-dimensional similarity method. These predictions were used only to identify target classes that may have functionally relevant counterparts in *B. bovis*, rather than to infer direct parasite targets. Because solute carrier family 2 (SLC2) glucose transporters dominated the prediction results, *B. bovis* hexose transporter 1 (BbHT1) was selected for exploratory analysis. In addition to glucose transporters, several kinase-associated human targets were predicted at lower ranks, supporting consideration of parasite kinases as an additional target class. *B. bovis* calcium-dependent protein kinase 4 (BbCDPK4) was therefore selected as a parasite kinase hypothesis based on the biological importance and pharmacological tractability of apicomplexan calcium-dependent protein kinases, rather than as a direct orthologue of a specific predicted human kinase.

#### 4.7.3. Comparative Protein Structure Modelling

ColabFold and AlphaFold2 [29,30] was used to model BbHT1 (UniProt S6B368; 472 amino acids),with the inward-open human Glucose transporter 1(GLUT1) structure (PDB 6THA) supplied as a custom template. GLUT1 was used because it is a well structurally characterized SLC2-family glucose transporter and SLC2 transporters dominated the ligand-based target-prediction results [31]. BbHT1 and the template shared approximately 29% sequence identity. Predictions used a subsampled multiple-sequence alignment with max_seq = 8 and max_extra_seq = 3220 recycles, four seeds, dropout enabled and Amber relaxation. Candidate models were ranked using local pocket pLDDT and the Alpha-carbon root-mean-square deviation (Cα RMSD) of conserved cavity-lining residues relative to the template. BbCDPK4 (RefSeq XP_001609485.1; 517 amino acids), on the other hand was modelled using the bumped kinase inhibitor (BKI)-bound *Plasmodium falciparum* CDPK4 structure (PDB 4QOX) as a custom template because it represents a homologous apicomplexan CDPK4 structure in an inhibitor-bound kinase conformation [32]. The target and template shared approximately 64% sequence identity. Predictions used max_seq = 512, max_extra_seq = 1024, six recycles, dropout disabled and Amber relaxation. Models were ranked by ATP-pocket pLDDT and examined for the small gatekeeper residue, the DFG motif and the conserved catalytic Lys–Glu ion pair.

#### 4.7.4. Ligand Preparation and Molecular Docking

Three three-dimensional conformers of DDI-1101229, designated representative conformers 1–3, were generated using the Experimental-Torsion Knowledge Distance Geometry (ETKDG) method and minimized using the Merck Molecular Force Field 94 (MMFF94) force field [33]. Each conformer was docked independently. Compound **49**, a reported human GLUT1 inhibitor and BKI-1294, a bumped kinase inhibitor with reported activity against apicomplexan CDPK-type kinases, were prepared identically and used only as comparative ligands for BbHT1 and BbCDPK4, respectively. GNINA was used to perform docking, with an exhaustiveness of 16 [34]. The BbHT1 search space was a 22 Å cubic box centered at (−0.235, 2.974, 0.196), corresponding to the predicted central cavity. The BbCDPK4 search space was a 20 Å cubic box centered at (6.418, −7.976, 6.889), corresponding to the ATP-binding pocket. For each target, the conformer producing the highest convolutional neural network (CNN) pose score was selected for interaction analysis and figure preparation. The Vina-style affinity of the displayed pose and the best affinity found among all poses were reported separately. Protein–Ligand Interaction Profiler (PLIP) 2.4.0 [35] was used to assign protein–ligand contacts. Molecular figures were prepared using Protein Imager 2.0 and PyMOL 3.1 (Schrödinger, LLC, New York, NY, USA). Published evidence was used to contextualize BbHT1 [21], BbCDPK4 [36], BKI-1294 [13] and compound **49** [37]. Compound **49** and BKI-1294 were treated as docking comparators and not as validated inhibitors of the corresponding *B. bovis* proteins.

## 5. Conclusions

This study applied a SYBR Green I-based high-throughput screening approach to a 9600-compound library and identified three inhibitors of *B. bovis*: DDI-1101229, DDI-8231037 and DDI-15106103. These compounds showed sub-micromolar in vitro activity, acceptable preliminary selectivity and synergistic interactions with DA, thus highlighting their potential as antibabesial candidates. Among them, DDI-8231037 and DDI-1101229 showed the most favorable overall profiles, combining potent activity with suitable predicted drug-like and pharmacokinetic properties. To our knowledge, these compounds have not been previously reported to possess antibabesial activity, highlighting their novelty as candidate chemotypes for further investigation. Although further studies are required to evaluate their in vivo efficacy, pharmacokinetic properties, toxicity, mechanisms of action and activity against DA-reduced-susceptibility parasites, these findings provide hit compounds that may serve as starting points for the development of improved treatments against bovine babesiosis.

## Figures and Tables

**Table 1:** In vitro anti-*B. bovis* activity, cytotoxicity, and SI of tested compounds.

Compound Structures	Compound ID	IC_50_ (µM)	CC_50_ (µM)	SI
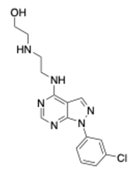	DDI-1101229	0.27 ± 0.15	>10	>37.0
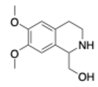	DDI-8231037	0.31 ± 0.06	>10	>33.3
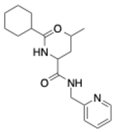	DDI-1441015	1.65 ± 0.64	>10	>6.1
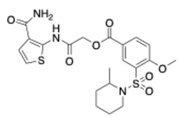	DDI-15106103	0.68 ± 0.23	>10	>14.7
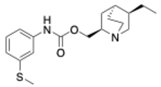	DDI-226297	1.42 ± 1.88	>10	>7.2
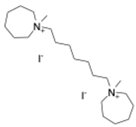	DDI-1423810	1.91 ± 1.24	>10	>5.2
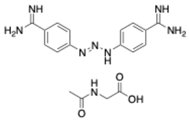	DA	0.24 ± 0.05	>10	>41.2

IC_50_, 50% inhibitory concentration; CC_50_, 50% cytotoxic concentration; SI, selectivity index calculated as CC_50_/IC_50_. Data are presented as mean ± SD from three independent experiments.

**Table 2:** Synergistic interaction of selected compounds with diminazene aceturate against *B. bovis*.

Drug Combination	CI (Fa = 0.5)	Interpretation
DDI-1101229 + DA	0.3 ± 0.2	Synergy
DDI-8231037 + DA	0.3 ± 0.1	Synergy
DDI-15106103 + DA	0.4 ± 0.2	Synergy

Combination index (CI) values were calculated at the ED_50_ level using the Chou–Talalay method. Fa represents the fraction affected; therefore, Fa = 0.5 corresponds to 50% inhibition, equivalent to the ED_50_ level. CI < 1.0 indicates synergistic interaction. DA, diminazene aceturate; ED_50_, 50% effective dose; Fa, fraction affected; CI, combination index.

## Data Availability

The original contributions presented in this study are included in the article/Supplementary Materials. Further inquiries can be directed to the corresponding author.

## Supplementary Materials

The following supporting information can be downloaded at: https://www.mdpi.com/article/10.3390/ph19091464/s1, Figure S1: Correlation between parasitemia and fluorescence signal. Figure S2: Dose–response curves of selected hit compounds against *B. bovis*. Figure S3: Microscopic confirmation of reduced *B. bovis* parasitemia. Table S1. In silico ADME and drug-likeness properties of selected compounds. Table S2. Target Prediction for DDI-1101229. Table S3. Comparative modelling and binding-site quality of BbHT1 and BbCDPK4. Table S4. Conformer-level GNINA results for DDI-1101229. Table S5. Docking and interaction summary for DDI-1101229. Table S6. Molecular docking results against BbHT1: compound DDI-1101229 (best conformer) versus control inhibitor 49. Table S7. Molecular docking results against BbCDPK4: compound DDI-1101229 (best conformer) versus bumped kinase inhibitor 1294.

## Abbreviations

aa	Amino acids
ADME	Absorption, distribution, metabolism and excretion
AMED	Japan Agency for Medical Research and Development
ATP	Adenosine triphosphate
BCDIN3D	BCDIN3 domain-containing RNA methyltransferase
BbCDPK4	*Babesia bovis* calcium-dependent protein kinase 4
BbHT1	*Babesia bovis* hexose transporter 1
BKI	Bumped kinase inhibitor
CCK-8	Cell Counting Kit-8
CC_50_	50% cytotoxic concentration
CDPK	Calcium-dependent protein kinase
CI	Combination index
CLogP	Calculated logarithm of the octanol/water partition coefficient
CNN	Convolutional neural network
CV	Coefficient of variation
CYP	Cytochrome P450
CYP1A2	Cytochrome P450 1A2
CYP3A4	Cytochrome P450 3A4
DA	Diminazene aceturate
DDI	Drug Discovery Initiative
DFG	Aspartate–phenylalanine–glycine
DNA	Deoxyribonucleic acid
ED_50_	50% effective dose
EDTA	Ethylenediaminetetraacetic acid
EGFR	Epidermal growth factor receptor
ELQ	Endochin-like quinolone
ERBB2	Erb-b2 receptor tyrosine kinase 2
ETKDG	Experimental-Torsion Knowledge Distance Geometry
Fa	Fraction affected
GI	Gastrointestinal
GLUT1	Glucose transporter 1
HTS	High-throughput screening
IC_50_	50% inhibitory concentration
IMDP	Imidocarb dipropionate
JSPS	Japan Society for the Promotion of Science
KAKENHI	Grants-in-Aid for Scientific Research
MDBK	Madin–Darby bovine kidney
MMFF94	Merck Molecular Force Field 94
MMV	Medicines for Malaria Venture
MTOR	Mechanistic target of rapamycin
PAE	Predicted aligned error
PAINS	Pan-assay interference compounds
PDB	Protein Data Bank
PfCDPK4	*Plasmodium falciparum* calcium-dependent protein kinase 4
PIK3CA	Phosphatidylinositol-4,5-bisphosphate 3-kinase catalytic subunit alpha
PIP4K2A	Phosphatidylinositol-5-phosphate 4-kinase type 2 alpha
PLIP	Protein–Ligand Interaction Profiler
pLDDT	Predicted Local Distance Difference Test
R^2^	Coefficient of determination
RBC	Red blood cell
RefSeq	Reference Sequence
RMSD	Root-mean-square deviation
ROP18	Rhoptry protein 18
RPS6KB1	Ribosomal protein S6 kinase B1
S/B	Signal-to-background ratio
SD	Standard deviation
SI	Selectivity index
SLC2	Solute carrier family 2
SLC2A1	Solute carrier family 2 member 1
SLC2A2	Solute carrier family 2 member 2
SLC2A3	Solute carrier family 2 member 3
SMILES	Simplified molecular-input line-entry system
TPSA	Topological polar surface area
Tris-HCl	Tris(hydroxymethyl)aminomethane hydrochloride
WISE	World-leading Innovative and Smart Education
