# Supplementary material for: Identification of Small-Molecule Inhibitors of Babesia bovis by High-Throughput SYBR Green I Screening
Source: Pharmaceuticals (Basel). 2026 Sep 16;19(9):1464. doi: 10.3390/ph19091464 (PMC13610274; doi:10.3390/ph19091464)
Supplement: Supplementary file 1 [file pharmaceuticals-19-01464-s001.zip › Figure S2.pdf]

DDI-1101229

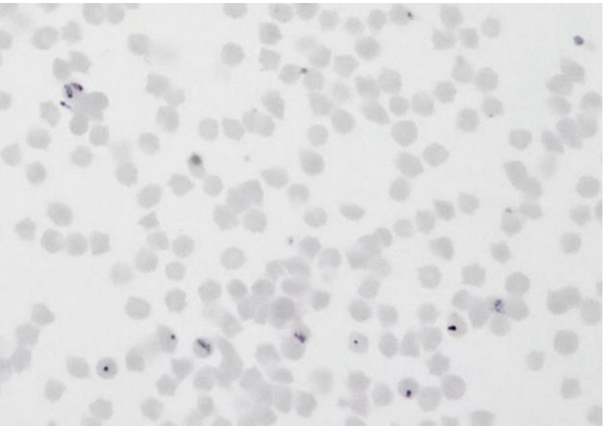

DDI-8231037

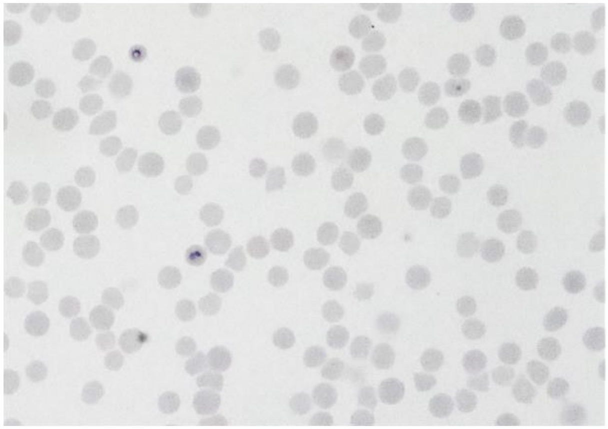

DDI-1441015

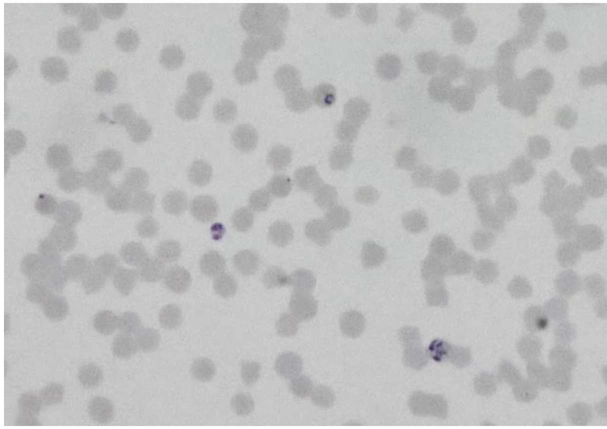

DDI-15106103

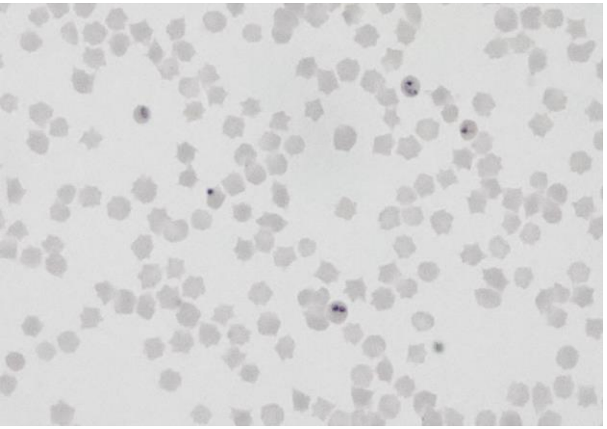

DDI-226297

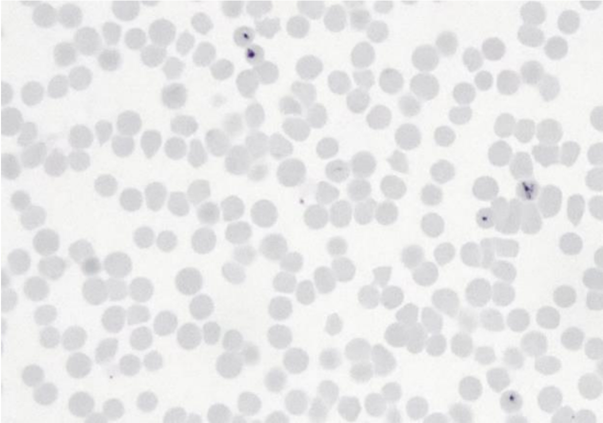

DDI-1423810

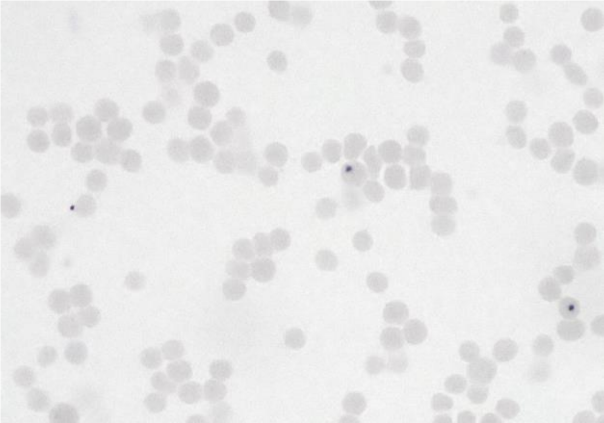

Untreated infected control

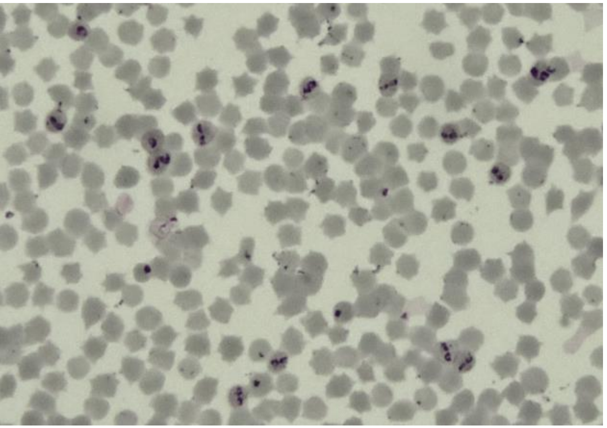

Figure S2. Microscopic confirmation of reduced *B. bovis* parasitemia. Representative Giemsa-stained thin blood smears of untreated infected *B. bovis* cultures and cultures treated with selected compounds at 2 µM. Parasitemia was quantified by microscopic counting of infected erythrocytes. At 2 µM, parasitemia was reduced to 1.9%, 1.3%, 2.0%, 3.7%, 2.9%, and 4.8% in cultures treated with DDI-1101229, DDI-8231037, DDI-1441015, DDI-15106103, DDI-226297, and DDI-1423810, respectively, compared with 5.4% in untreated infected controls.
