# Supplementary material for: Identification of Small-Molecule Inhibitors of Babesia bovis by High-Throughput SYBR Green I Screening
Source: Pharmaceuticals (Basel). 2026 Sep 16;19(9):1464. doi: 10.3390/ph19091464 (PMC13610274; doi:10.3390/ph19091464)
Supplement: Supplementary file 1 [file pharmaceuticals-19-01464-s001.zip › Figure S3.pdf]

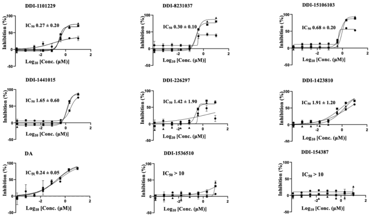
DDI-1101229
IC50 0.27 ± 0.20
DDI-8231037
IC50 0.30 ± 0.10
DDI-15106103
IC50 0.68 ± 0.20
DDI-1441015
IC50 1.65 ± 0.60
DDI-226297
IC50 1.42 ± 1.90
DDI-1423810
IC50 1.91 ± 1.20
DA
IC50 0.24 ± 0.05
DDI-1536510
IC50 > 10
DDI-154387
IC50 > 10
Inhibition (%)
Log10 [Conc. (μM)]
