# Supplementary material for: Identification of Small-Molecule Inhibitors of Babesia bovis by High-Throughput SYBR Green I Screening
Source: Pharmaceuticals (Basel). 2026 Sep 16;19(9):1464. doi: 10.3390/ph19091464 (PMC13610274; doi:10.3390/ph19091464)
Supplement: Supplementary file 1 [file pharmaceuticals-19-01464-s001.zip › supplimentary_tables.pdf]

Table S1. ***In silico*** **ADME and drug-likeness properties of selected compounds**

Parameter	DDI-8231037	DDI-1101229	DDI-15106103	DA	IMDP
Molecular Weight (g/mol)	223.0	346.8	495.6	398.42	496.56
Consensus LogP	1.1	1.4	2.8	1.3	1.9
TPSA (Å²)	50.7	86.8	182.1	202.89	164.51
GI Absorption	High	High	Low	Low	Low
CYP Inhibition	None	CYP1A2 inhibitor	CYP3A4 inhibitor	None	None
Lipinski	0 violations	0 violations	0 violations	2 violations: N or O > 10; NH or OH > 5	2 violations: N or O > 10; NH or OH > 5
Veber	0 violations	0 violations	1 violation: TPSA > 140	1 violation: TPSA > 140	1 violation: TPSA > 140
PAINS	0 alerts	0 alerts	0 alerts	1 alert: azo A	0 alert
Bioavailability Score	0.6	0.6	0.6	0.2	0.2

DA, diminazene aceturate; IMDP, imidocarb dipropionate; TPSA, topological polar surface area; GI, gastrointestinal; CYP, cytochrome P450; PAINS, pan-assay interference compounds.

Table S2. Target Prediction for DDI-1101229

Rank	Predicted human target	Gene	UniProt	Target class	Probability
1	Solute carrier family 2 member 3	SLC2A3	P11169	Electrochemical transporter	0.799
2	Solute carrier family 2 member 1	SLC2A1	P11166	Electrochemical transporter	0.799
3	Solute carrier family 2 member 2	SLC2A2	P11168	Electrochemical transporter	0.749
4	Epidermal growth factor receptor	EGFR	P00533	Receptor tyrosine kinase	0.387
5	Phosphatidylinositol-5-phosphate 4-kinase type 2α	PIP4K2A	P48426	Transferase	0.357
6	Ribosomal protein S6 kinase β1	RPS6KB1	P23443	Kinase	0.305
7	Serine/threonine-protein kinase mTOR	MTOR	P42345	Kinase	0.274
8	PI3-kinase catalytic subunit α	PIK3CA	P42336	Transferase	0.274
9	RNA 5′-monophosphate methyltransferase	BCDIN3D	Q7Z5W3	Transferase	0.212
10	Receptor tyrosine-protein kinase ERBB2	ERBB2	P04626	Kinase	0.193

Predicted human targets for DDI-1101229 were obtained using SwissTargetPrediction with *Homo sapiens* selected as the target organism. Probability indicates the predicted likelihood of interaction with the corresponding target. UniProt, Universal Protein Resource; SLC2A1, solute carrier family 2 member 1; SLC2A2, solute carrier family 2 member 2; SLC2A3, solute carrier family 2 member 3; EGFR, epidermal growth factor receptor; PIP4K2A, phosphatidylinositol-5-phosphate 4-kinase type 2 alpha; RPS6KB1, ribosomal protein S6 kinase B1; MTOR, mechanistic target of rapamycin; PIK3CA, phosphatidylinositol-4,5-bisphosphate 3-kinase catalytic subunit alpha; BCDIN3D, BCDIN3 domain-containing RNA methyltransferase; ERBB2, erb-b2 receptor tyrosine kinase 2.

Table S3. Comparative modelling and binding-site quality of BbHT1 and BbCDPK4

Target	Sequence identifier	Template	Identity	Modelled state	Pocket pLDDT	Supporting structural observations
BbHT1	S6B368; 472 aa	Human GLUT1, PDB 6THA	0.29	Inward-open	91.4	Pocket PAE 2.56; conserved-cavity anchor RMSD 2.96 Å
BbCDPK4	XP_001609485.1; 517 aa	PfCDPK4, PDB 4QOX	0.64	Active, DFG-in	89.1	Thr141 gatekeeper; Asp208–Phe209–Gly210; Lys93 Glu112 distance 3.57 Å

Sequence identifiers, template structures, sequence identity, modelled conformational state, pocket pLDDT scores, and key structural features used to assess model suitability for exploratory molecular docking are shown for each target.

Table S4. Conformer-level GNINA results for DDI-1101229

Target	Conformer	Highest-CNN-pose affinity (kcal/mol)	Maximum CNN pose score	Best affinity among nine poses (kcal/mol)
BbHT1	rep1	−7.09	0.499	−8.21
BbHT1	rep2	−7.86	0.835	−8.26
BbHT1	rep3	−7.13	0.700	−8.26
BbCDPK4	rep1	−7.20	0.906	−7.20
BbCDPK4	rep2	−7.12	0.942	−7.31
BbCDPK4	rep3	−7.19	0.960	−7.32

rep1–rep3, representative conformers 1–3 of DDI-1101229; BbHT1, *Babesia bovis* hexose transporter 1; BbCDPK4, *Babesia bovis* calcium-dependent protein kinase 4; CNN, convolutional neural network. Highest-CNN-pose affinity refers to the affinity of the pose with the highest CNN pose score for each conformer, whereas best affinity represents the most favorable Vina-style docking score among the nine generated poses. Affinity values are expressed in kcal/mol.

Table S5. Docking and interaction summary for DDI-1101229

Target	Comparative ligand	Highest-CNN-pose affinity (kcal/mol)	CNN pose score	Best affinity among nine poses (kcal/mol)	Principal predicted contacts
BbHT1	Compound 49 [19]	−9.99	0.665	−10.81	Phe19, Trp395, Phe411, Thr415, Trp419; π-stacking with Phe19 and Trp395
BbCDPK4	BKI-1294 [18]	−8.88	0.860	−9.38	Tyr144, Thr141, Val78, Lys93, Leu194, Ile207, Asp208, Phe209

BbHT1, *Babesia bovis* hexose transporter 1; BbCDPK4, *Babesia bovis* calcium-dependent protein kinase 4; BKI, bumped kinase inhibitor; CNN, convolutional neural network. Highest-CNN-pose affinity refers to the affinity of the pose with the highest CNN pose score, whereas best affinity represents the most favorable Vina-style docking score among the nine generated poses. Compound 49 and BKI-1294 were used as comparative ligands for BbHT1 and BbCDPK4, respectively. Principal predicted contacts were identified using PLIP. Affinity values are expressed in kcal/mol.

Table S6. Molecular docking results against BbHT1: compound DDI-1101229 (best conformer) versus control inhibitor 49

Parameter	DDI-1101229 (rep2)	49 (control)
Best affinity (kcal/mol)	−8.26	−10.36
Top CNN pose score	0.835	0.672
CNN affinity	5.95	6.92
H-bond contacts	Ser123, Gly124, Tyr146	Trp395
Hydrophobic/π contacts	Phe19, Tyr146, Pro147, Trp395,Phe411	Phe19, Ile74, Ala127, Ile150, Phe386, Trp395, Phe411, Thr415, Trp419
π-stacking	—	Phe19, Trp395
Shared contacts with control	Phe19, Trp395, Phe411	—

Docking affinity is reported in kcal/mol; more negative values indicate stronger predicted binding. CNN, convolutional neural network; the CNN pose score reflects confidence in the predicted binding pose, whereas CNN affinity estimates binding strength. Hydrophobic/π contacts include nonpolar and aromatic interactions with the indicated BbHT1 residues. π-stacking denotes aromatic stacking interactions between the ligand and the indicated residues. Shared contacts are residues interacting with both DDI-1101229 and control inhibitor 49. —, no corresponding interaction/contact identified.

Table S7. Molecular docking results against BbCDPK4: compound DDI-1101229 (best conformer) versus bumped kinase inhibitor 1294

Parameter	DDI-1101229 (rep3)	1294 (BKI control)
Best affinity (kcal/mol)	−7.19	−8.88
Top CNN pose score	0.96	0.86
CNN affinity	6.82	7.76
Hinge H-bond	Tyr144	Tyr144
Other H-bond contacts	Leu70, Glu148	Glu148, Glu191
Gatekeeper contact	—	Thr141
Hydrophobic contacts	Val78, Leu194, Ile207	Leu70, Val78, Lys93, Leu116, Thr141, Glu148, Glu191, Leu194, Ile207, Asp208, Phe209
DFG-motif contact	—	Asp208, Phe209
Shared contacts with control	Tyr144, Glu148, Val78, Leu194, Ile207	—

Docking affinity is reported in kcal/mol; more negative values indicate stronger predicted binding.CNN, convolutional neural network; the CNN pose score reflects confidence in the predicted binding pose, whereas CNN affinity estimates binding strength. BKI, bumped kinase inhibitor. DFG, Asp-Phe-Gly motif. Shared contacts are residues interacting with both DDI-1101229 and control inhibitor 1294. —, no corresponding interaction/contact identified.
