# Supplementary material for: Identification of Small-Molecule Inhibitors of Babesia bovis by High-Throughput SYBR Green I Screening
Source: Pharmaceuticals (Basel). 2026 Sep 16;19(9):1464. doi: 10.3390/ph19091464 (PMC13610274; doi:10.3390/ph19091464)
Supplement: Supplementary file 1 [file pharmaceuticals-19-01464-s001.zip › Figure S1.pdf]

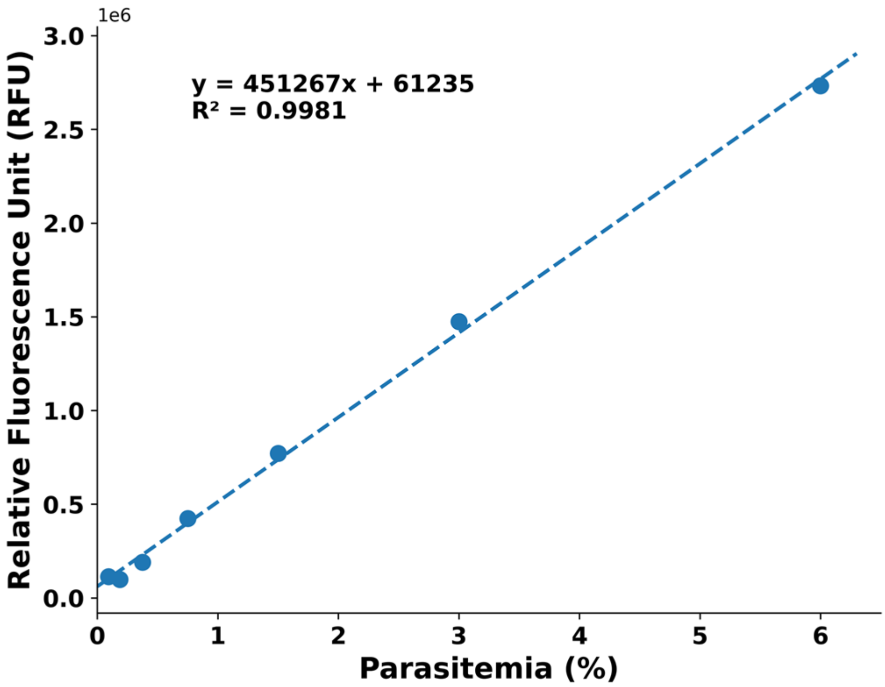

Figure S1. Correlation between parasitemia and fluorescence signal.

Relative fluorescence units (RFU) were measured across increasing parasitemia levels to assess the linearity and robustness of the fluorescence-based assay. The assay showed a strong positive linear relationship between parasitemia and fluorescence intensity, with a regression equation of $y = 451267x + 61235$ and an R^2 value of 0.9981, demonstrating that fluorescence signal reliably reflected parasite burden across the tested range.
